# Comparison of host cell gene expression in cowpox, monkeypox or vaccinia virus-infected cells reveals virus-specific regulation of immune response genes

**DOI:** 10.1186/1743-422X-10-61

**Published:** 2013-02-20

**Authors:** Daniel Bourquain, Piotr Wojtek Dabrowski, Andreas Nitsche

**Affiliations:** 1 Centre for Biological Threats and Special Pathogens 1, Robert Koch Institute, Nordufer 20, Berlin 13353, Germany; 2 Central Administration 4 (IT), Robert Koch Institute, Nordufer 20, Berlin 13353, Germany

**Keywords:** Orthopoxvirus, Cowpox virus, Vaccinia virus, Monkeypox virus, Microarray, Gene expression, Host cell response

## Abstract

**Background:**

Animal-borne orthopoxviruses, like monkeypox, vaccinia and the closely related cowpox virus, are all capable of causing zoonotic infections in humans, representing a potential threat to human health. The disease caused by each virus differs in terms of symptoms and severity, but little is yet know about the reasons for these varying phenotypes. They may be explained by the unique repertoire of immune and host cell modulating factors encoded by each virus. In this study, we analysed the specific modulation of the host cell’s gene expression profile by cowpox, monkeypox and vaccinia virus infection. We aimed to identify mechanisms that are either common to orthopoxvirus infection or specific to certain orthopoxvirus species, allowing a more detailed description of differences in virus-host cell interactions between individual orthopoxviruses. To this end, we analysed changes in host cell gene expression of HeLa cells in response to infection with cowpox, monkeypox and vaccinia virus, using whole-genome gene expression microarrays, and compared these to each other and to non-infected cells.

**Results:**

Despite a dominating non-responsiveness of cellular transcription towards orthopoxvirus infection, we could identify several clusters of infection-modulated genes. These clusters are either commonly regulated by orthopoxvirus infection or are uniquely regulated by infection with a specific orthopoxvirus, with major differences being observed in immune response genes. Most noticeable was an induction of genes involved in leukocyte migration and activation in cowpox and monkeypox virus-infected cells, which was not observed following vaccinia virus infection.

**Conclusion:**

Despite their close genetic relationship, the expression profiles induced by infection with different orthopoxviruses vary significantly. It may be speculated that these differences at the cellular level contribute to the individual characteristics of cowpox, monkeypox and vaccinia virus infections in certain host species.

## Background

Viruses of the family *Poxviridae* are characterized by their large and complex virions, a double-stranded DNA genome of 130–375 kbp and the cytosol as the place of replication [1]. As a family, poxviruses are able to infect both vertebrate and invertebrate hosts. Poxviruses of vertebrates are divided into ten genera [2]. Out of these, especially the genus orthopoxvirus (OPV) contains several important pathogens of humans and animals, including some zoonotic members [3]. After eradication of variola virus (VARV) [4], the most common OPV infections are caused today by monkeypox virus (MPXV), vaccinia virus (VACV) and cowpox virus (CPXV) [3]. VACV is the prototype member of the OPV genus and the best-studied one. VACV served as vaccine during the smallpox eradication campaign and its virulence in man is generally low. However, several severe complications have been reported to occur after vaccination or laboratory-acquired exposition [5,6]. Furthermore, researchers in Brazil have been reporting several cases of naturally occurring zoonotic VACV infections since 1999 [7-10]. Similar to VACV infections, human CPXV infections of healthy individuals are generally self-limiting and cause only localised skin lesions. However, severe generalised CPXV infections with lethal outcome have been reported in immunocompromised patients [11,12]. In Europe and parts of northern and central Asia, endemic CPXV are the most common cause of human OPV infections [13]. It is assumed that wild rodents serve as reservoir hosts for CPXV. However, transmission to various other species including several domestic and zoo animals has been reported, and of all OPV CPXV is suggested to possibly infect the widest range of host species [3]. To date, no direct human-to-human transmission has been reported [13,14]. In contrast to VACV and CPXV, MPXV causes a disease resembling smallpox in humans, but with milder morbidity and lower mortality rates [15]. The severity of the disease depends on the geographic origin of the different MPXV strains, as virus isolates from Central Africa have been shown to be more virulent than those from Western Africa [16,17]. MPXV was first described as an illness of captive zoo monkeys [18], and rodents are assumed to be the natural host [3].

Concerning the potential threat arising from VACV, MPXV and CPXV, a deepening of our knowledge about the mechanisms underlying differences in poxviral pathogenesis and species-specificity would allow greatly improved risk assessment.

Today, more than 100 OPV genomes have been fully sequenced. Therefore the unique arsenal of viral genes encoded by each virus is often known, and in several cases detailed information about viral gene functions is also available. However, current knowledge about corresponding events in the host cell and especially of the differences in host response towards infection and host modulation by these viruses is still limited. Several studies described the transcriptional host response towards infection of different cell types with VACV or closely related rabbitpox virus, either using microarrays or ultrahigh-throughput DNA sequencing for genome-wide transcriptome analysis [19-25]. These studies report that host genes are predominantly downregulated during infection, which may be due to an unspecific suppression of host mRNAs by VACV. The fewer cellular genes which are specifically induced by infection are supposed to play key roles in viral replication or host response to infection, respectively [19,22,25]. Especially mRNAs which are associated with the NF-κB cascade, apoptosis, signal transduction and ligand-mediated signalling were reported to be induced in response to VACV infection [21]. Similarly, MPXV was described to cause a decrease in host mRNA levels, accompanied by an increase of fewer mRNAs following infection of MK2 cells [26]. Besides these studies, which focused on analysis of the host response to infection by one specific virus, Rubins et al. directly compared how VACV and MPXV alter the gene expression programs in their hosts [27]. However, to our knowledge, so far no study has investigated the transcriptional response of the host cell towards CPXV infection. As CPXV encodes several unique genes, not to be found in the VACV or MPXV genome, we decided to compare the way how these zoonotic poxviruses alter the gene expression of their host cells. We chose HeLa cells as a model system and characterized changes in the host transcriptional programs in response to infection with CPXV, MPXV or VACV, respectively, using microarrays representing the whole human genome. We aimed to identify mechanisms that are either common to OPV infection or to specific OPV species at the cellular level, allowing a more detailed description of differences in virus-host cell interactions between individual OPV.

## Results

### Experimental design

HeLa cells were infected with either CPXV reference strain Brighton Red (BR), the highly pathogenic central African MPXV strain MSF-6 [28] or the mouse-pathogenic VACV strain IHD-W [29]. HeLa cells were chosen because of their high susceptibility towards OPV infection and because many fundamental studies on OPV biology and on host gene expression changes following infection have been performed with this cell line [19-21,23,25]. Cells were infected at a high multiplicity of infection of 5 plaque forming units (PFU) per cell to guarantee synchronous infection of all cells. Total RNA from infected and mock-infected control cells was isolated at 6 h post infection. This point of time was chosen to allow enough time for establishment of infection and progression to late viral gene expression but to avoid the risk of cell lysis and RNA degradation after completion of the first replication cycle [1,24,30]. Furthermore, previous studies described that specific gene activation in response to infection mainly occurs until 6 h post infection, while unspecific downregulation of genes prevails at later stages of infection [19,23]. Therefore, we chose 6 h post infection to analyse the specific host cell modulation by CPXV, MPXV and VACV and to analyse differences in the cellular response towards these viruses. The successful infection of HeLa cells with all viruses and the uniform progression of infection were confirmed by immunofluorescence microscopy of cells infected in parallel. At 24 h post infection with each virus nearly all cells were infected and showed a pronounced cytopathic effect (data not shown). To verify that all viruses had uniformly proceeded from intermediate to late viral transcription at 6 h post infection, mRNA expression of the late transcription factor VLTF-1 (VACCP-G8R) and of the late 11 kDa virion core protein (VACCP-F17R) was confirmed via quantitative real-time PCR (data not shown).

Total RNA from infected and mock-infected control cells was used for cRNA microarray analysis of host cell transcription. All experiments were performed in duplicate using RNA samples from two independently infected cell cultures for each analysis. To compare the gene expression profiles, ratios were calculated by dividing the merged normalised signal intensities of infected samples by mock-control signal intensities. Genes that exhibited a fold change (FC) in gene expression ≥2 and signal intensities that were significantly above the background with p-values ≤0.01 were chosen for further analysis (see also Additional file 1).

### The majority of host genes remains unaffected after infection

**Figure 1:**
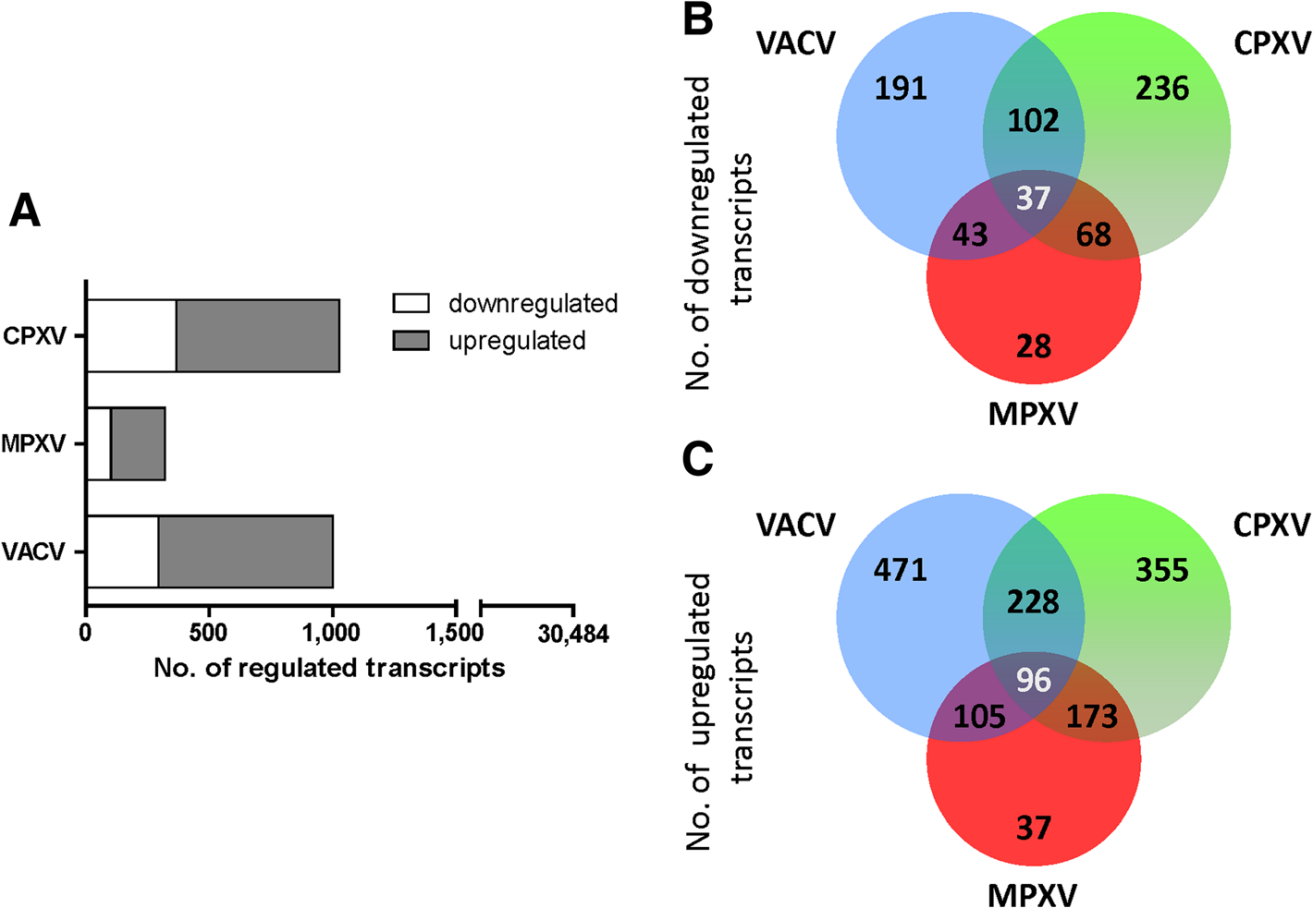
**Number of host mRNAs regulated by CPXV, MPXV or VACV infection.** Shown are the numbers of cellular transcripts that exhibited expression changes larger than 2-fold after infection with each virus in two independent microarray experiments. Transcripts that were upregulated after infection in comparison to non-infected cells are shown in dark grey, transcripts that were downregulated in white (**A**). Intersections of transcript downregulation (**B**) or upregulation (**C**) in response to VACV, CPXV or MPXV infection are shown in the Venn diagram.

At first, we compared the gene expression data of each virus-infected sample to the expression profile of the mock-infected HeLa cells to determine the relative modulation of cellular transcription induced by infection with CPXV, MPXV or VACV, respectively. As shown in Figure 1A, a majority of 96% of the assayed cellular transcripts remained unchanged after infection with either virus strain. Out of the 30,484 individual genes and transcripts assayed in total (using 41,000 probes), 1,027 (3.7%) transcripts in CPXV-infected cells, only 321 (1.1%) transcripts in MPXV-infected cells and 1,002 (3.3%) transcripts in VACV-infected cells showed expression changes larger than 2-fold after infection. Among the 1,027 CPXV-modulated transcripts, 660 (64.3%) were upregulated and only 367 downregulated. A similar distribution can be seen following MPXV infection which resulted in upregulation of 219 (68.2%) and downregulation of 102 transcripts. Likewise, VACV infection resulted in upregulation of 708 (70.7%) and downregulation of 294 transcripts. Taken together, these data show that an infection with CPXV, VACV and especially MPXV induces only marginal changes of the cellular gene expression profile at 6 h post infection.

### Identification of gene expression changes common to CPXV, MPXV and VACV infection

Despite of the fact that the number of host cell transcripts affected by CPXV and VACV infection was similar, the number of genes regulated by CPXV and VACV was only 330 (~33%) of approximately 1,000. Among the 321 host transcripts which are modulated by MPXV infection, 241 (75.1%) were also affected by CPXV but only 148 (46.1%) by VACV infection. Beyond that, by comparing all three data sets, we could identify a cluster of 133 transcripts whose expression is modulated after infection with each virus. Out of these, 96 transcripts (72.2%) were upregulated in all virus-infected samples and 37 were downregulated (Figure 1B, C). We applied a Gene Ontology (GO) term-based cluster analysis of these 133 transcripts to identify the biological processes in which the genes are involved. Analysis of gene set enrichment was used to identify statistically overrepresented processes. This analysis revealed that the most prominent group were genes involved in chromatin organisation, containing 50 of the 133 transcripts. Out of these, 46 belonged to the histone family of proteins. Increased detection of histone mRNAs following OPV infection has been described before to be an experimental artefact caused by *de novo* polyadenylation of these mRNAs by the viral poly(A) polymerase, resulting in their enhanced amplification and detection [22,27]. Therefore histone mRNAs were excluded from further analysis, resulting in 87 genes which were regulated by infection with CPXV, MPXV as well as VACV. A comparison of the gene expression of these genes is shown in Additional file 2. Besides the histone family members, further functional clusters of transcripts were identified.

### Analysis of commonly regulated genes reveals an induction of epidermal growth factor family members and genes involved in regulation of MAPK activity by CPXV, MPXV and VACV

Cluster analysis of the 87 non-histone transcripts that were affected by all OPV tested revealed a statistically significant (Bonferroni-corrected group p-value = 1.1 × 10^-5^) overrepresentation of genes involved in inactivation of mitogen-activated protein kinase (MAPK) activity (DUSP5/6, SPRED1/2, SPRY2/4). All of these genes were upregulated in virus-infected cells and all of them are known to be induced following MAPK-ERK activation [31-35]. In this context, we could also observe a strong upregulation of the early growth-response gene EGR1 and a less pronounced upregulation of EGR2 by all three viruses. EGR1 upregulation via the MAPK-ERK pathway following VACV and CPXV infection has been described before by Silva et al. [36]. Here we show a similar effect following MPXV infection as well. To further evaluate the general impact of OPV infection on biological processes of the infected cell, we examined potential enrichment of these 87 commonly affected genes in known canonical pathways, according to the KEGG pathway database. We could identify 12/87 genes which significantly mapped to two distinct pathways, the ErbB pathway with 5.75% of associated genes (AREG, CBLB, EREG, HBEGF, PRKCB; p = 1.6 × 10^-3^) and the JAK-STAT pathway with 4.52% of associated genes (CBLB, IL4R, LIF, SPRED1/2, SPRY2/4; p = 2.1 × 10^-4^). The ErbB signalling pathway comprises a family of receptor tyrosine kinases which are coupling the binding of extracellular growth factor ligands to intracellular signalling pathways such as the MAPK pathway. In this context, upregulation of the epidermal growth factor (EGF) family members EREG, AREG and HBEGF, respectively, may be seen as a trigger for enhanced MAPK-ERK signalling which itself results in upregulation of DUSP5/6, SPRED1/2, SPRY2/4 and EGR1/2, respectively, as a feedback mechanism. The induction of EGF family members and genes involved in regulation of MAPK activity by VACV, CPXV as well as MPXV is summarized in Table 1. Interestingly, only the immune-regulatory CBLB gene and the multifunctional PRKCB gene were downregulated by infection.

**Table 1:** Induction of EGF family members and genes involved in regulation of MAPK activity by CPXV, MPXV and VACV

	**Gene**	**Gene expression changes (average FC)**^**a**^		
		**CPXV**	**MPXV**	**VACV**
**MAPK activity**	DUSP5	6.6	4.9	3.6
	DUSP6	8.1	9.5	5.2
	EGR1	14.3	30.8	98.6
	EGR2	2.6	5.1	4.2
	IL4R	6.2	5.8	2.9
	LIF	9.7	12.6	4.6
	SPRY1	12.7	n.s.^b^	n.s.^b^
	SPRY2	7.7	7.1	7.0
	SPRY4	20.8	30.1	11.8
	SPRED1	2.8/3.7^c^	2.9/3.0^c^	2.2/2.2^c^
	SPRED2	2.0	2.3	2.8/7.8^c^
**both**	CBLB	−2.7	−3.4	−2.5
**ErbB pathway**	AREG	10.2	8.3	4.8
	EREG	4.4/6.7^c^	7.0/10.3^c^	2.9/3.0^c^
	HBEGF	4.0/5.4^c^	2.3/2.6^c^	2.1/2.8^c^
	PRKCB	−4.9	−3.4	−2.4

^a^ Cluster and pathway analysis of the 87 non-histone transcripts that were affected by all OPV tested revealed a statistically significant overrepresentation of genes involved in inactivation of mitogen-activated protein kinase (MAPK) activity and growth factor signalling. Shown are gene expression changes of the identified genes at 6 h post infection in CPXV-, VACV- or MPXV-infected cells in comparison to mock-infected cells as average fold change (FC) values.

^b^ Values that did not meet the analysis cut-off of an average FC ≥2.0 and p ≤0.05 are designated n.s. (not significant).

^c^ Two values separated by a slash designate values obtained from two different probes on the microarray.

### Identification of virus-specific changes in gene expression induced by CPXV, MPXV or VACV infection

In contrast to these few commonly regulated genes, most infection-regulated genes differ from virus to virus. These differences were most prominent when comparing CPXV or MPXV to VACV infection. To this end, we attributed the enrichment of all genes to biological processes that were differentially regulated following infection in comparison to non-infected cells. This analysis again shows the common regulation of the MAP kinase pathway by all three viruses, but also highlights major differences in the gene clusters affected by CPXV, MPXV or VACV infection. All results are summarized in Table 2. In the case of CPXV infection we could identify 16 overrepresented clusters, including several genes encoding protein kinases, proteins mediating kinase activity and blood vessel development. However, this cluster also contained a significant overrepresentation of genes which can be involved in immune response-activating signal transduction such as Toll-like receptor signalling pathways. Notably, other significantly overrepresented groups were associated with immune system processes, too, such as regulation of leukocyte migration and myeloid cell differentiation.

**Table 2:** Cluster analysis of infection-induced gene expression changes

	**Leading term**^**a**^	**No. of genes**^**b**^	**Group p-value**	**Term p-value**^**b**^
**CPXV**	blood vessel development	49	4.8 × 10^-11^	7.6 × 10^-9^
	negative regulation of protein kinase activity	25	1.4 × 10^-14^	1.1 × 10^-8^
	myeloid cell differentiation	22	6.5 × 10^-4^	5.4 × 10^-3^
	negative regulation of protein serine/threonine kinase activity	22	8.0 × 10^-18^	1.1 × 10^-10^
	ERK1 and ERK2 cascade	20	1.7 × 10^-11^	3.8 × 10^-6^
	regulation of ERK1 and ERK2 cascade	19	1.8 × 10^-12^	1.3 × 10^-6^
	regulation of actin cytoskeleton organization	18	1.1 × 10^-4^	4.5 × 10^-3^
	negative regulation of MAP kinase activity (gene set 1)	17	4.4 × 10^-8^	1.0 × 10^-8^
	negative regulation of MAP kinase activity (gene set 2)	17	1.4 × 10^-11^	1.0 × 10^-8^
	regulation of leukocyte migration	14	6.2 × 10^-8^	5.5 × 10^-5^
	negative regulation of hormone secretion	13	4.6 × 10^-5^	1.0 × 10^-6^
	negative regulation of Wnt receptor signalling pathway	11	4.2 × 10^-3^	1.1 × 10^-1^
	nephron development	11	2.4 × 10^-5^	3.8 × 10^-2^
	positive regulation of nitric oxide biosynthetic process	10	5.0 × 10^-6^	2.5 × 10^-5^
	positive regulation of transmission of nerve impulse	9	7.7 × 10^-4^	3.9 × 10^-3^
	positive regulation of mitosis	7	2.2 × 10^-6^	1.8 × 10^-2^
	negative regulation of insulin receptor signalling pathway	6	n.a.^c^	1.3 × 10^-2^
**MPXV**	positive regulation of NF-κB transcription factor activity	12	1.8 × 10^-16^	5.3 × 10^-8^
	negative regulation of intracellular protein kinase cascade	11	n.a.^c^	2.6 × 10^-6^
	negative regulation of hormone secretion	10	n.a.^c^	3.3 × 10^-9^
	negative regulation of MAP kinase activity	8	n.a.^c^	1.8 × 10^-5^
	cellular response to lipopolysaccharide	8	9.3 × 10^-9^	8.1 × 10^-5^
	positive regulation of Rho GTPase activity	7	n.a.^c^	2.7 × 10^-4^
	astrocyte differentiation	6	n.a.^c^	2.0 × 10^-4^
	regulation of gliogenesis	6	n.a.^c^	1.3 × 10^-3^
**VACV**	negative regulation of MAPK cascade	12	7.0 × 10^-6^	2.5 × 10^-4^
	ovum-producing ovary development	8	n.a.^c^	4.8 × 10^-2^
	potassium ion transmembrane transport	6	n.a.^c^	4.1 × 10^-2^
	cellular potassium ion transport	6	n.a.^c^	4.1 × 10^-2^

^a^ Overrepresented biological processes identified by GO clustering of the genes affected by CPXV, MPXV or VACV infection. Related terms were merged into functional groups to reduce redundancy, and the most significant term of the group was defined as group-leading term.

^b^ The degree of connectivity between terms is calculated using κ statistics, and the calculated κ score is also used for defining functional groups. Shown are the identified overrepresented processes, the number of infection-regulated genes assigned to that process and the term p-values.

^c^*n*.*a*. = not applicable. Only term p-values are given if no grouping was done.

In the case of MPXV infection again an enrichment of genes involved in negative regulation of MAPK activity and negative regulation of intracellular protein kinase cascade was observed. Similar to CPXV infection, immunity-associated clusters were likewise overrepresented. This included the biological processes “cellular response to lipopolysaccharide” and “positive regulation of NF-κB transcription factor activity”. Many of the regulated genes within either of these processes are also involved in positive regulation of leukocyte migration, Toll-like receptor signalling and chemotaxis.

In contrast, host genes modulated by VACV infection showed a significant overrepresentation only in four Gene Ontology (GO) terms. The most significantly affected biological process was negative regulation of intracellular protein kinase cascade and especially of the MAPK cascade. However, in comparison to CPXV and MPXV infection, no immune response-specific processes could be identified. Taken together, we could identify considerable differences concerning the expression of immunity-associated host genes which were significantly affected by CPXV and MPXV but not by VACV infection.

### Analysis of canonical pathways reveals a significant influence of CPXV and MPXV but not VACV infection on cellular pathways involved in the immune response and infectious diseases

**Figure 2:**
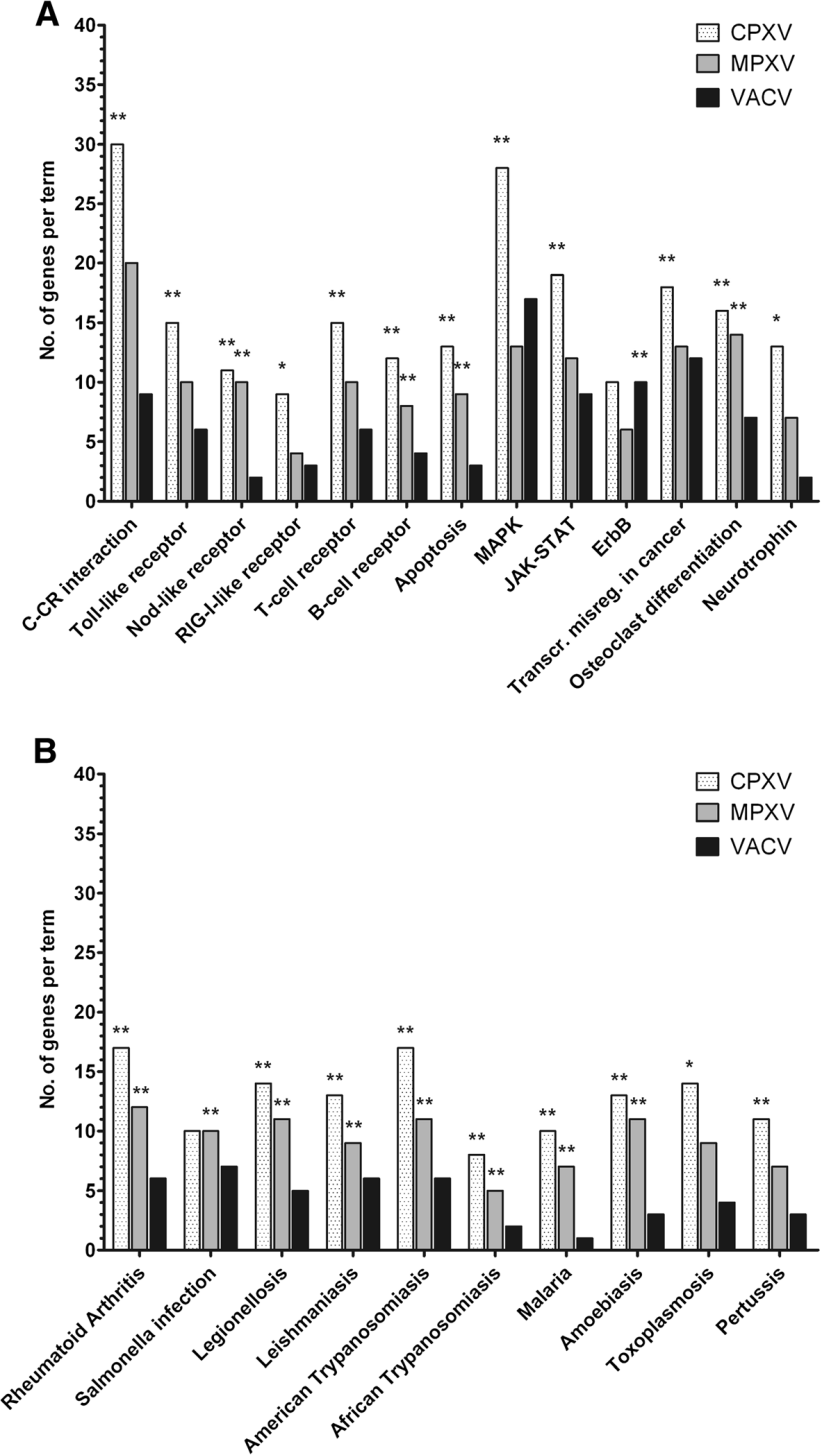
**CPXV- and MPXV- but not VACV-modulated cellular pathways are involved in infectious diseases.** Cellular pathways that were significantly affected by infection with CPXV, MPXV or VACV, respectively, as indicated by * (p ≤ 0.05) or ** (p ≤ 0.01). Along with pathways which describe general processes of the cell, we identified several immune system-specific pathways (**A**) and several pathways which are specific to certain diseases (**B**). P-values indicate the probability of random association between the genes in the data set and the canonical pathway. Abbreviations used in the figure: C-CR interaction = cytokine–cytokine receptor interaction; Transcr. misreg. in cancer = transcriptional misregulation in cancer.

Infection with CPXV influenced the expression of 1,027 genes, 244 of which were represented in the KEGG pathway database, and 126 of these genes could be mapped to 21 significantly affected pathways (Figure 2). In the case of MPXV infection 104 genes were found in KEGG, and 35 of them could be mapped to pathways that met the criteria. Both after CPXV and MPXV infection a noticeable overrepresentation of immunity-related pathways was observed, along with a significant overrepresentation of genes mapping to pathways specific for certain infectious diseases. Interestingly, for the most part the disease-specific pathways which were affected by CPXV and MPXV infection describe infections caused by intracellular pathogens or inflammatory processes. For VACV-infected cells 191 out of 949 genes were found in KEGG. However, in sharp contrast to the numerous cellular pathways affected by CPXV or MPXV infection, only the ErbB signalling pathway was significantly affected by VACV infection. All significantly affected pathways are shown in Figure 2.

Taken together, this shows the general importance of growth factor and MAPK signalling for OPV infection. Furthermore, the analysis highlighted that immune response-associated signalling pathways are highly affected by CPXV and MPXV, but not by VACV infection. Several genes which are affected by CPXV and MPXV infection were involved in disease-specific pathways. However, the majority of those genes also mapped to other, less pathogen-specific pathways like Toll-like and NOD-like receptor signalling pathways, phagosome, MAPK signalling and apoptosis pathways, albeit with a less significant enrichment.

### CPXV and MPXV modulate genes involved in leukocyte migration and TLR signalling

We analysed enrichment of genes involved in immune system processes to further specify these results. To this end, we matched these genes against the GO immune system process database. We could identify an overrepresentation of genes involved in positive regulation of leukocyte migration (p = 1.2 × 10^-2^, 21.8%) and Toll-like receptor 2 signalling pathways (p = 4.9 × 10^-2^, 18.1%) after CPXV infection, and in positive regulation of leukocyte migration (p = 6 × 10^-3^, 12.7%) and TRIF-dependent Toll-like receptor signalling pathways (p = 5.9 × 10^-3^, 11.3%) after MPXV infection. No such overrepresentation could be detected among genes that showed regulation following VACV infection (data not shown).

### CPXV and MPXV strongly induce genes involved in chemotaxis or leukocyte activation

**Figure 3:**
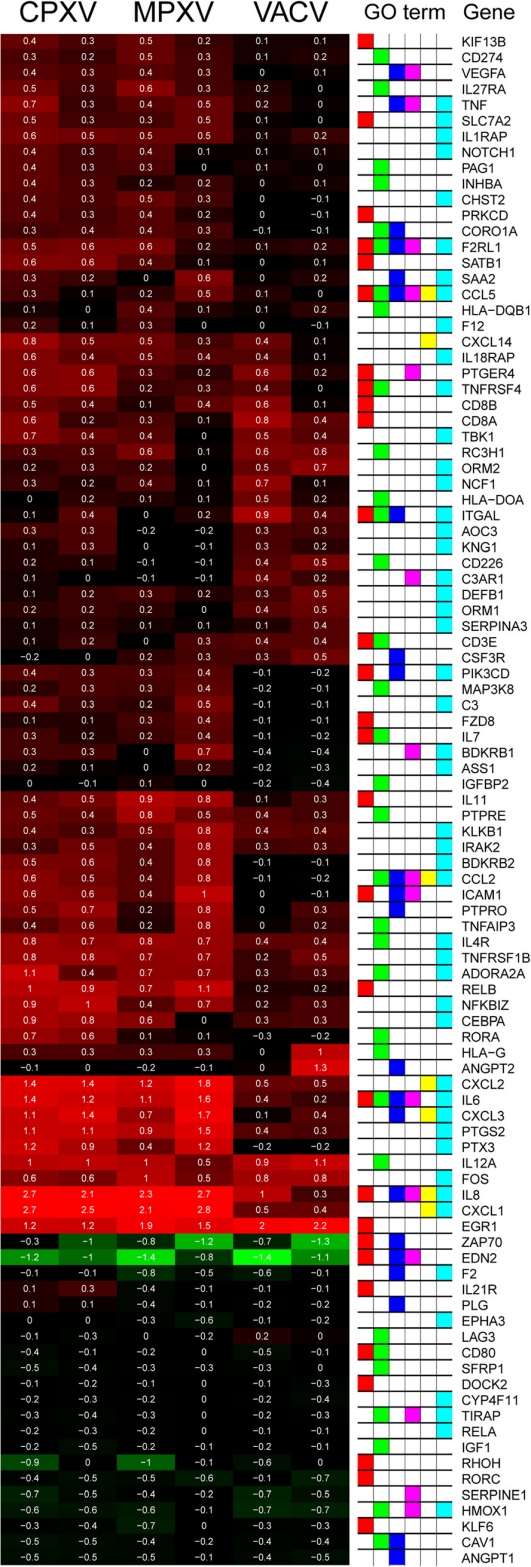
**Modulation of immune response-associated genes by CPXV, MPXV or VACV infection.** An overview of the changes in host gene expression patterns of immune response-associated genes induced by all three different OPV. Folds of change in gene expression in comparison to mock-infected cells are represented as gradient red and green colour representing low and high relative expression in the respective microarray (shown are 2 arrays per virus). Numbers indicate the log10 of the change fold values. GO term assignment of the respective genes is displayed by different colours. Red = “leukocyte activation”, green = “regulation of leukocyte activation”, blue = “leukocyte migration”, magenta = “regulation of leukocyte migration”, yellow = “human chemokine activity”, turquoise = “inflammatory response”. Only those genes are shown which had at least a 2-fold change in expression relative to the mock-infected control and for which the direction of regulation detected by both arrays was the same. Additionally, regulation in each selected gene differs by a factor of at least 2 between at least two of the samples.

A comparison of the changes in the host gene expression of immune response-affiliated genes, as defined by respective GO terms, showed several genes which were highly upregulated after CPXV or MPXV but not or only slightly upregulated after VACV infection. Especially following CPXV infection an upregulation of several inflammatory genes was observed. Furthermore, several genes encoding proteins which possess chemokine activity or are involved in positive regulation of leukocyte migration were induced by CPXV infection. This upregulation was similar for MPXV infection, but not or only weakly present following VACV infection. Additionally, we could observe a pronounced upregulation of several genes involved in leukocyte activation and positive regulation of leukocyte activation following CPXV infection, which could not be observed following VACV infection and only to a lesser extent following MPXV infection (Figure 3). The numbers of up- and downregulated genes which are associated with these GO terms are also shown in Table 3.

**Table 3:** Numbers of at least 2-fold up- or downregulated immune response-associated genes following CPXV, MPXV or VACV infection grouped by associated GO term

	**Leukocyte activation**		**Regulation of leukocyte activation**		**Leukocyte migration**		**Regulation of leukocyte migration**		**Human chemokine activity**		**Inflammatory response**	
	**Up**	**Down**	**Up**	**Down**	**Up**	**Down**	**Up**	**Down**	**Up**	**Down**	**Up**	**Down**
CPXV	17	6	15	6	12	4	8	4	6	0	29	2
MPXV	16	6	16	2	13	6	9	3	7	0	31	3
VACV	9	5	11	4	6	5	4	4	5	0	20	4

Taken together, these data indicate that in contrast to VACV infection the regulation of immune response-associated genes by CPXV and MPXV infection may more likely indicate a pronounced antiviral immune response of the host cell than a process beneficial for viral replication. This is supported by the observed upregulation of many pro-inflammatory genes and especially chemokine genes following CPXV and MPXV infection.

## Discussion

In this study we applied a comparative analysis of changes of the host cell gene expression profile following infection with CPXV, MPXV or VACV, respectively. We addressed the question whether the different characteristics of each virus, especially the distinctive repertoire of host modulating factors encoded by each virus, may in part be reflected by different characteristics of the infected cells. OPV are well known to suppress the antiviral host defence, exploit the host cell machinery for reproduction and to inhibit or delay cell death. However, most of our knowledge about these mechanisms originates from studies using VACV. More recently, after the US outbreak in 2003, MPXV has come into focus [37,38], but again, our knowledge about the differences in virus–host interactions between VACV and MPXV and even more so between VACV, MPXV and CPXV is still limited. In this study we investigated the host-cell transcriptome of CPXV-, MPXV- or VACV-infected cells to explore the different capabilities of each virus to interact with the host cell.

We found the host cell transcriptome to be mainly unaffected by poxviral infection, despite the major morphological changes induced by infection. Although this holds true for OPV infection in general, the impact of MPXV infection on the transcriptome seems to be uniquely low. This might indicate an even more elaborate “stealth” program than that performed by VACV or CPXV, allowing MPXV to especially well avoid the responses of the innate and adaptive immune systems to the developing infection. It would be interesting to investigate if this is true for MPXV infection in general, or if it might be a characteristic of the highly pathogenic central African MPXV strains in contrast to less pathogenic west African strains [17].

The data presented are in agreement with the results by Rubins et al. which show no general decline in cellular mRNAs after infection of HeLa cells with MPXV (Zaire strain) or VACV (Western Reserve strain) [27]. In our study we could prove that the same was true for CPXV infection. Other studies describe that the cellular gene expression is generally suppressed in response to infection and that only few genes are specifically induced [19,23,26]. In our study, we found that more than two thirds of the few host cell transcripts which exhibited regulation in response to infection were upregulated at 6 h post infection. However, we cannot exclude the possibility of a more pronounced downnregulation of host cell genes at later stages of infection. Furthermore, in our study, only genes were taken into account that exhibited at least 2-fold change in gene expression, to set the focus on genes that exhibit stronger regulation. Therefore, a mild global downregulation of cellular genes which was below the cut-off value might have been neglected (see also Additional file 3). However, while repression of host genes by OPV is thought to be generalised and likely nonspecific, cellular genes which are induced by infection are of particular interest, as they are thought to play key roles in viral replication or host response to infection [19,22,25].

We found the infection-induced changes of the gene expression profiles of CPXV-, MPXV- and VACV-infected cells to be largely different. However, we could still identify several cellular transcripts which showed similar modulation after infection with each virus, the most significant one being histone mRNAs. An apparent induction of histone genes has been described previously in other studies [19-21,26,27]. However, this is thought to be an experimental artefact caused by *de novo* polyadenylation of the histone transcripts by the viral poly(A) polymerase, as histone mRNAs are the only mRNAs in eukaryotes that lack a poly(A) tail in general [22,27]. This is supported by the fact that Yang et al. reported histone mRNAs to be overrepresented at 4 h post VACV infection even after poly(A)-specific mRNA isolation procedures [21]. If this was the case, amplification of histone mRNAs from infected samples would be greatly enhanced by the poly(A)-dependent procedure we used. We therefore decided to exclude histone mRNAs from further analysis.

After exclusion of histone mRNAs, we could identify 87 cellular transcripts which seemed to be commonly modulated by OPV infection in general. Most noticeable was an apparent infection-induced upregulation of genes involved in inactivation of MAPK activity. The upregulated Sprouty (SPRY2/4) and Sprouty-related (SPRED1/2) proteins are known to be induced by growth factor receptor activation-mediated MAPK-ERK activation via Ras as a self-regulatory feedback inhibition mechanism [31-33]. Similarly, the upregulated DUSP5/6 and EGR1/2 genes are commonly induced as early response genes after activation of the MAPK-ERK signalling pathway and act as negative regulators of ERK phosphorylation [34,35]. Therefore we suggest that the observed upregulation of genes involved in inactivation of MAPK activity may reflect a feedback mechanism towards virus-induced stimulation of MAPK-ERK activity, probably via an enhanced virus-induced signalling through EGF receptors. This theory is supported by the observed overrepresentation of components of the ErbB pathway among the commonly affected transcripts and by the upregulation of EGF family members following infection. Although modulation of the MAPK-ERK pathway seems to be a common effect of OPV infection, its importance differs from virus to virus. Silva et al. showed that inhibition of MEK/ERK signalling resulted in a significant decrease in VACV yield, but had no impact on CPXV replication [36,39,40]. The importance of MAPK-ERK signalling in the context of MPXV replication has not been addressed yet.

Despite these few commonly affected gene sets, major differences in the transcriptional response towards infection with CPXV, MPXV or VACV prevailed. Most interesting was an enrichment of genes involved in immunity-associated processes and pathways in CPXV- and MPXV-infected cells, which was strikingly absent following VACV infection. This included especially genes that are involved in leukocyte migration and Toll-like receptor signalling, which seemed to be affected by CPXV as well as by MPXV infection.

Interestingly, we could identify several pathways specific to certain infectious diseases to be affected by CPXV and MPXV infection. As most of these pathways were specific to infection by intracellular pathogens, this result may be explained by universal mechanisms of host modulation and inflammatory host response which could be affected alike by these diseases and by CPXV and MPXV.

A large proportion of the immunity-associated genes which were affected by CPXV and MPXV consisted of pro-inflammatory cytokine genes and genes involved in leukocyte chemotaxis or activation of immune cells. As most of these genes showed pronounced upregulation after CPXV and MPXV infection, this might reflect an inadequate subversion of the hosts’ antiviral response. This may be supported by the fact that an induction of genes implicated in the immune response, e.g. IL6, could also be observed in response to attenuated modified VACV Ankara infection of HeLa cells [19] but not in response to non-attenuated VACV WR infection [23]. Interestingly, an induction of pro-inflammatory cytokines and chemokines in response to CPXV and MPXV infection was observed by other studies as well. Increased IL8 gene expression following MPXV infection of MK2 cells was reported by Alkhalil et al. [26]. And *in vivo*, strong secretion of IL-6, IL-8, and G-CSF or of IL-6, IL-8 and CCL-2, respectively, was observed following infection of cynomolgus macaques (*Macaca fascicularis*) with MPXV or CPXV, respectively [41-43].

## Conclusion

To our knowledge, this study represents the first description of changes of the host cell gene expression program in response to infection with CPXV, a virus that is circulating in Europe as well as in parts of Asia and that displays some individual features in the genus OPV. While the host cell response towards VACV infection has been analysed in great detail by previous studies, we describe one of the first attempts to directly compare the impact of CPXV, MPXV and VACV on the gene expression profile of the host cell. We could show a major non-responsiveness of the transcriptional program of the host cell towards infection with all three viruses, which may be a sign of successful virostealth. In spite of this, we could also identify several genes which seemed to be affected by OPV infection in general or exhibited regulation by infection with a specific virus. By assigning these genes to certain biological processes or pathways, we could show that CPXV and MPXV infection induces several genes which are involved in immunity. This may indicate that CPXV and MPXV infection, but not VACV infection, induce a pronounced inflammatory response which may result in attraction of leukocytes. This leads to the question whether the induction of those genes is a part of the antiviral activity of the host or a process in benefit of infection as a mechanism that facilitates viral spread. The latter may be suggested by the important role which infected leukocytes are known to play in OPV dissemination across the body [27,44]. It will be interesting to analyse the effect of different viral strains or cell types on the observed effects, especially concerning the large genetic diversity of different CPXV strains or of VACV strains from different geographical origin.

## Methods

### Cells and culture conditions

All cell lines were obtained from American Type Culture Collection (ATCC, Manassas, VA). HeLa cells (ATCC ID CCL-2) were cultivated in Eagle’s minimal essential medium (EMEM) supplemented with 10% heat-inactivated foetal calf serum (FCS, PAA) and 2 mM of L-glutamine (PAA). Hep-2 cells (ATCC ID CCL-23) and Vero E6 cells (ATCC ID CRL-1586) were both cultured in Dulbecco’s Modified Eagle Medium (DMEM) containing 10% FCS and 2 mM of L-glutamine. All cell lines were routinely screened for the absence of mycoplasma contamination.

### Viruses and infection conditions

VACV strain IHD-W (ATCC ID VR-1441, NCBI GenBank KC201194) and CPXV strain Brighton Red (ATCC ID VR-302; NCBI GenBank AF482758) were obtained from ATCC (ATCC, Manassas, VA). MPXV strain MSF-6 (sequence to be submitted soon), which was obtained from a fatally infected human in Congo, was kindly provided by Prof. Dr. Hermann Meyer (Institut für Mikrobiologie der Bundeswehr, München, Germany) [28]. All viruses were propagated in Hep-2 cells and cell culture supernatants which were clarified by centrifugation were used as virus stocks. The titres of virus stocks were determined by plaque assay [45] in Vero E6 cells as described before [46] and were shown to be comparable for CPXV, VACV and MPXV. All virus stocks were screened for absence of mycoplasma contamination. For infection experiments, HeLa cells were grown in 25 cm^2^ cell culture flasks (Nunc) and incubated overnight before infection with each virus at a multiplicity of infection of 5 PFU/cell. Mock infections were performed using culture medium free of any virus. After adsorption of virus for 1 h at 37°C, the virus-containing medium or mock medium was removed and cells were washed twice with phosphate buffered saline (PBS) to reduce side effects caused by biological factors probably present in the inoculum. Afterwards, fresh culture medium was added. Cells were then incubated at 37°C until 6 h post infection. All infection experiments were performed in biosafety level 3 (S3) laboratories in accordance with the German legal regulations.

### Sample acquisition and RNA preparation for microarray analysis

Total RNA was isolated using Trizol® Reagent (Invitrogen) as described in the manufacturer’s protocol for adherent cells. RNA samples from two independently infected cell cultures were used for each analysis. Human total RNA quality and integrity were determined using the Agilent RNA 6000 Nano Kit on the Agilent 2100 Bioanalyzer (Agilent Technologies). RNA was quantified by measuring A260 nm on the ND-1000 Spectrophotometer (NanoDrop Technologies). Sample labelling was performed as detailed in the Agilent “One-Color Microarray-Based Gene Expression Analysis” protocol (version 5.7, part number G4140-90040). Briefly, 1 μg of each total RNA sample was used for the amplification and labelling step, using the Agilent Quick Amp Labeling Kit (Agilent Technologies) in the presence of cyanine 3-CTP. Yields of cRNA and the dye-incorporation rate were measured with the ND-1000 Spectrophotometer (NanoDrop Technologies).

### Microarrays and hybridisation

The hybridisation procedure was performed according to the “One-Color Microarray-Based Gene Expression Analysis” protocol (Agilent Technologies, version 5.7, part number G4140-90040). Briefly, 1.65 μg of Cy3-labeled fragmented cRNA in hybridisation buffer was hybridised overnight (17 h, 65°C) to Agilent Whole Human Genome Oligo Microarrays 4 × 44 K, using Agilent’s recommended hybridisation chamber and oven. Following hybridisation, the microarrays were washed once with the Agilent Gene Expression Wash Buffer 1 for 1 min at room temperature, followed by a second wash with preheated Agilent Gene Expression Wash Buffer 2 (37°C) for 1 min. The last washing step was performed with acetonitrile.

### Scanning and data analysis

Fluorescence signals of the hybridised Agilent Microarrays were detected using Agilent’s Microarray Scanner System (Agilent Technologies). The Agilent Feature Extraction Software (FES) 10.5.1.1 was used to read out and process the microarray image files. For determination of differential gene expression, FES-derived output data files were further analysed using the Rosetta Resolver gene expression data analysis system (Rosetta Biosoftware, Rosetta error model [47]). Ratios were calculated by dividing sample signal intensity through control signal intensity. The signal intensities from the single-experiment raw data lists were normalised by dividing the intensity values by their median. Putative candidate genes were selected based on a minimum fold change (FC) ≥2 and p-value ≤0.01. The calculation of merged ratios for replicate experiments was performed by calculating pair-wise log-ratios and log-ratio error and the subsequent combination to one ratio in an error-weighted averaging procedure. Data visualisation was done via generation of heat maps from the normalised signal intensity data using the software environment R (v2.15.0).

### Pathway analysis

Analysis of enrichment of genes to certain canonical pathways was done using the software Cytoscape 2.8.2 in combination with the Cytoscape plugin ClueGO v1.4 [48,49]. Enrichment analysis was based on terms provided by the KEGG databases (received 7^th^ February 2012) [50]. The probability of random association between the genes in the data set and the canonical pathway was calculated with a right-sided minimum likelihood test on the hypergeometric distribution using the Bonferroni correction of p-values. Pathways that were identified with statistical support of p-values ≤0.05 were taken into account. Due to the multitude of pathways which were identified by this method when analysing all differentially regulated genes, we decided to focus on significantly overrepresented terms. These were characterized by p-values ≤0.05, a ratio of ≥10% of term-annotated genes and a minimum of at least 4 genes per term.

### Cluster analysis

Analysis of enrichment of genes to certain GO terms was also done using ClueGO. Enrichment analysis was based on terms provided by the GO (database file received 6^th^ February 2012) [51]. Again, we decided to focus on significantly overrepresented terms which were characterized by p-values ≤0.01, a ratio of ≥10% of term-annotated genes and a minimum of at least 6 genes per term. Furthermore, related terms were merged into functional groups and the most significant term of the group was defined as group leading term. The degree of connectivity between terms is calculated using κ statistics and the calculated κ score is also used for defining functional groups [48].

### Microarray data

Microarray data have been submitted to the Gene Expression Omnibus (GEO) and can be searched using the record ID: GSE36854.

## Competing interests

The authors declare that they have no competing interests.

## Authors’ contributions

DB was responsible for design, conduct and completion of this work as well as for data analysis and writing of this manuscript. PWD was responsible for data processing and statistical analysis of microarray data. AN was the principal investigator and was primarily responsible for all aspects of research design and coordination and contributed to drafting the manuscript. All authors read and approved the final manuscript.

## Supplementary Material

Additional file 1 **Overview of changes in transcript expression following infection.** Scatter plot of signal intensities of all spots. Data was obtained by merging the data sets of two replicates. The plots illustrate a comparison of signal intensities from non-infected cells versus CPXV- (**A**), MPXV- (**B**) or VACV- (**C**) infected cells. The signal intensities of each feature represented by a dot are shown in double logarithmic scale. X-axis: mock signal intensity; y-axis: infected sample signal intensity. Red diagonal lines define areas of 2-fold differential signal intensities. Blue spots define unchanged expression; red dots: transcripts significantly upregulated and green dots: transcripts significantly down-regulated in the infected samples (p-value ≤0.01). The grey cross in the legend marks the number of significantly up- and downregulated genes. Click here for file

Additional file 2 ** genes regulated by CPXV, MPXV and VACV.** Shown is the relative expression of genes which were regulated by infection with all OPV tested to a similar extent. Included are genes that exhibited more than 2-fold up- or downregulation following infection. Red and green colour displays up- or downregulation in the infected cells compared to non-infected cells (shown are 2 arrays per virus). Numbers indicate the log10 of the change fold values. Click here for file

Additional file 3 **Distribution of up- or downregulated genes.** The figure shows the distribution of up- or downregulated transcripts following infection with CPXV (**A**), MPXV (**B**) or VACV (**C**) in comparison to non-infected cells. The degree of regulation compared to the non-infected control is displayed on the x-axis. A global minor downregulation below the set cut-off value of ≥2 fold change occurred following infection with MPXV and VACV. Click here for file
