# Supplementary figures and images for: Comparison of host cell gene expression in cowpox, monkeypox or vaccinia virus-infected cells reveals virus-specific regulation of immune response genes
Source: Virol J. 2013 Feb 20;10:61. doi: 10.1186/1743-422X-10-61 (PMC3599072; doi:10.1186/1743-422X-10-61)

A

mock vs. CPXV

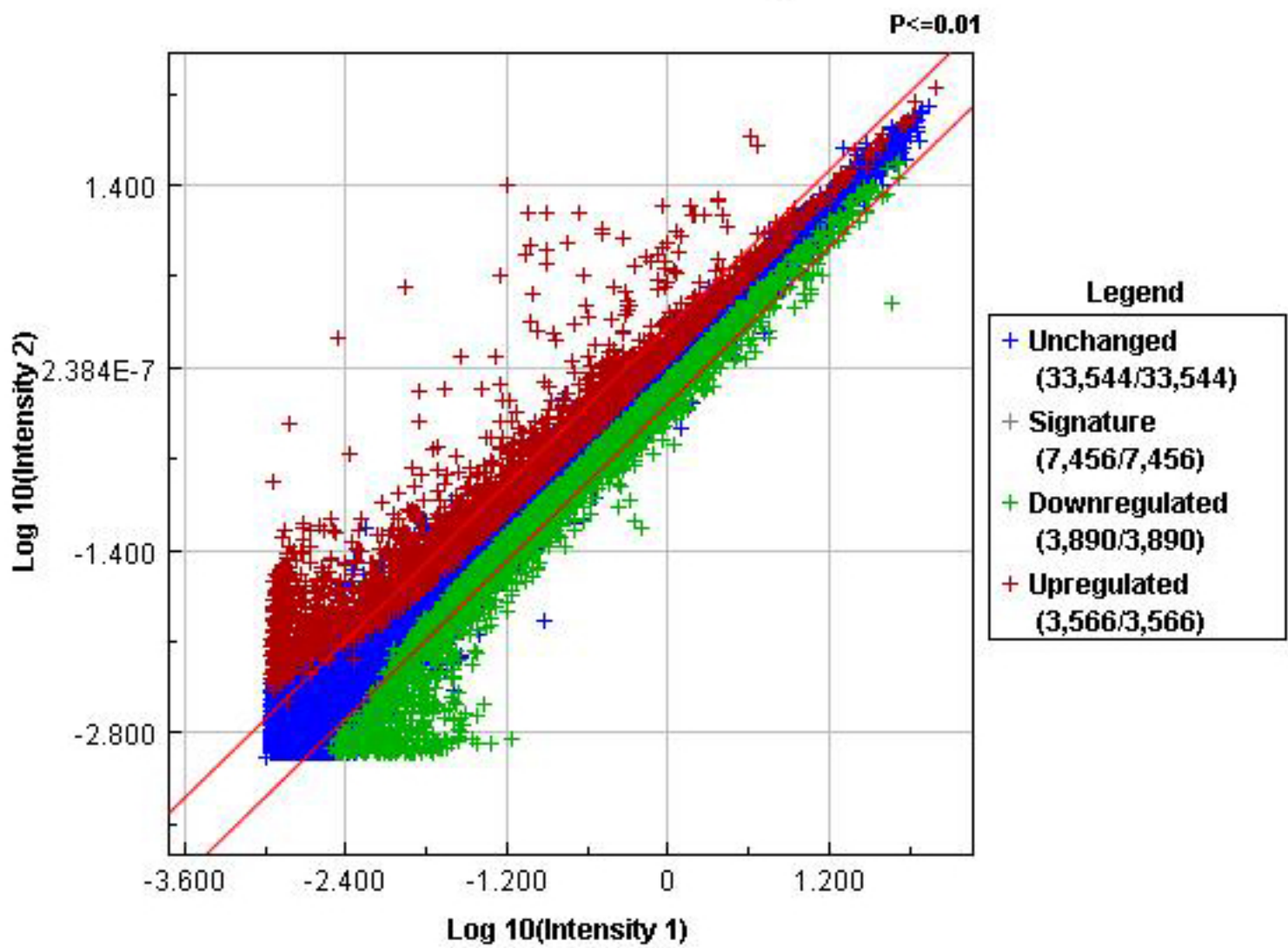

B

mock vs. MPXV

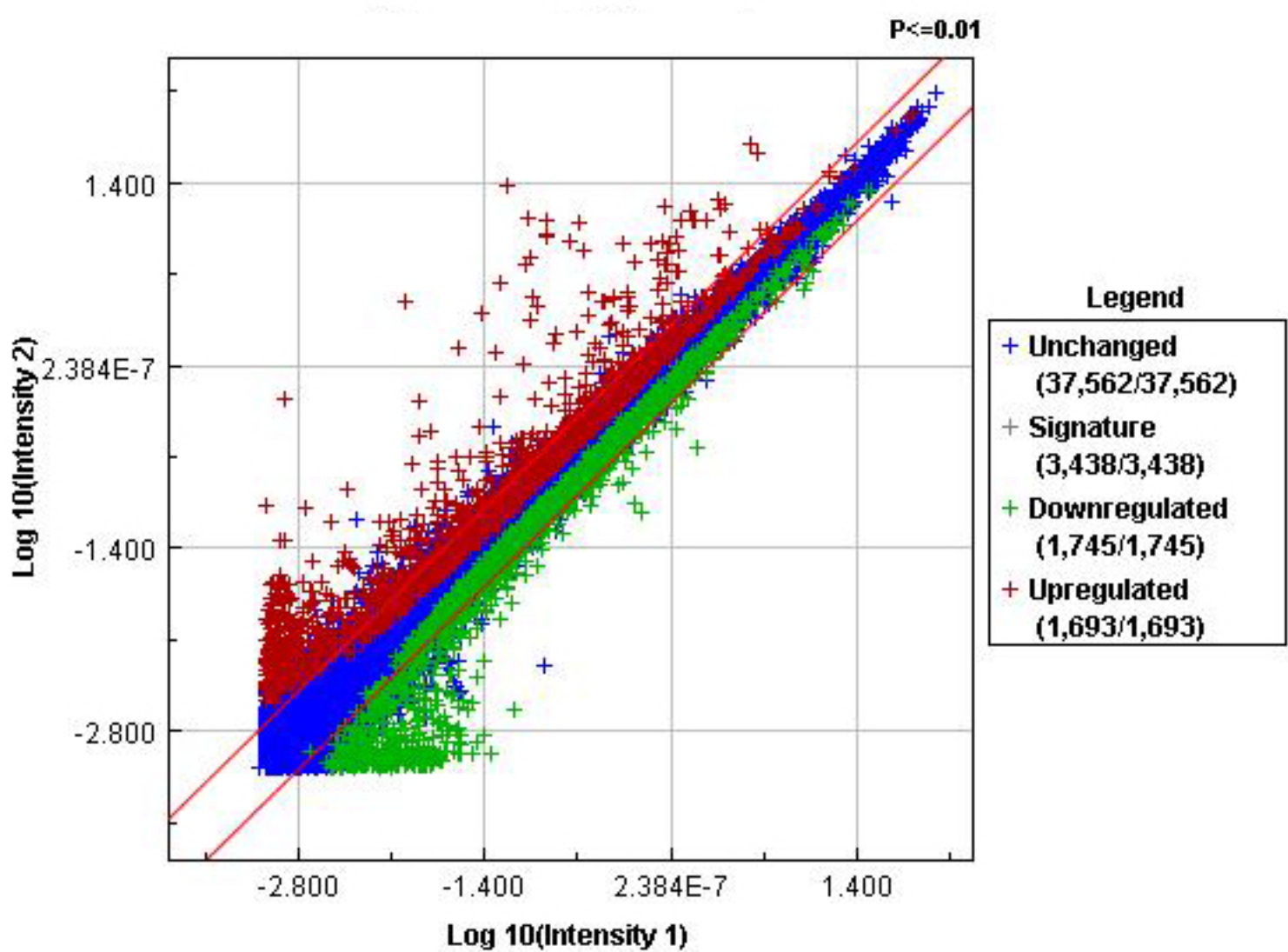

C

mock vs. VACV

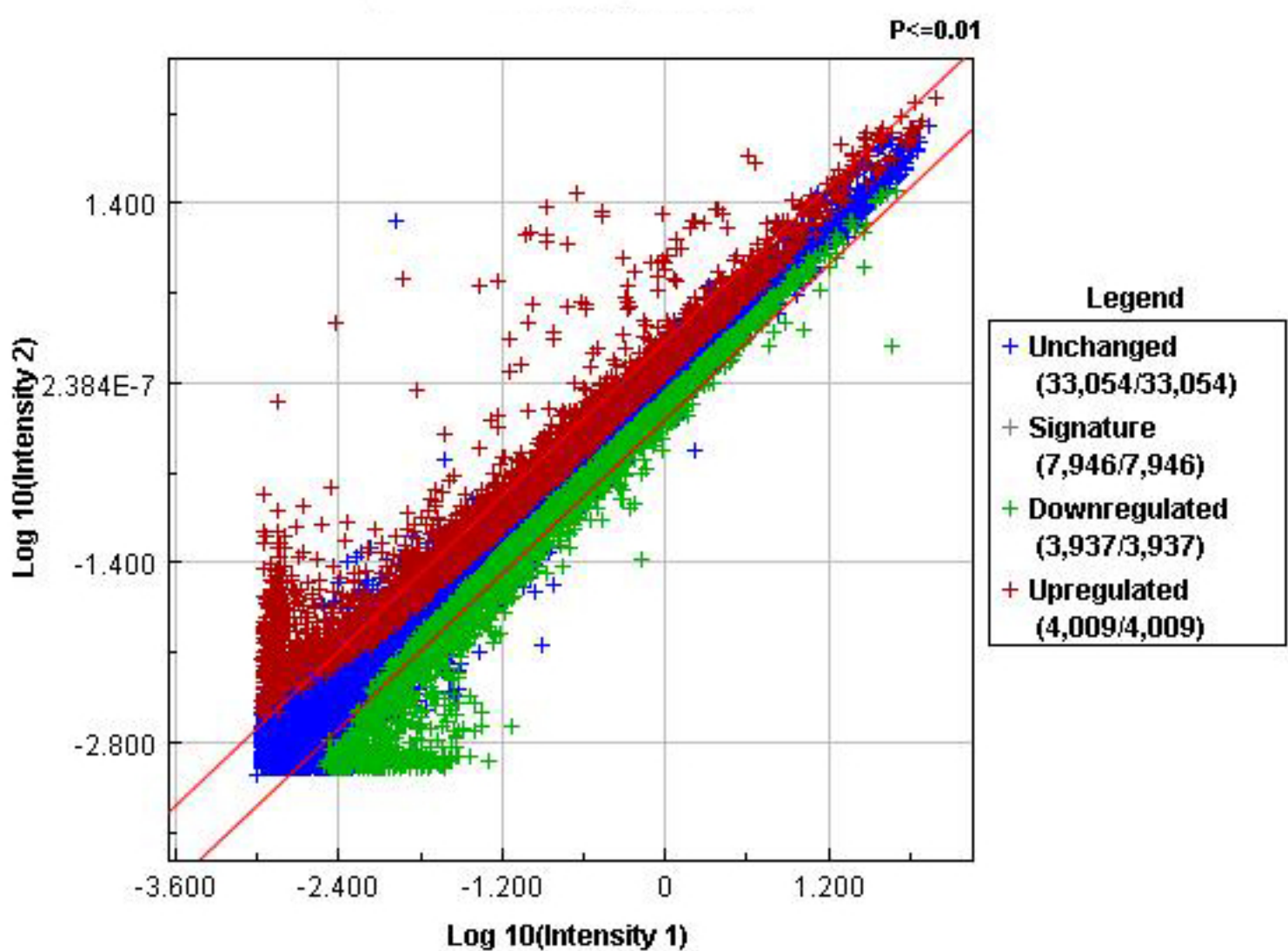

Supplement: Additional file 1 — Overview of changes in transcript expression following infection. Scatter plot of signal intensities of all spots. Data was obtained by merging the data sets of two replicates. The plots illustrate a comparison of signal intensities from non-infected cells versus CPXV- (A), MPXV- (B) or VACV- (C) infected cells. The signal intensities of each feature represented by a dot are shown in double logarithmic scale. X-axis: mock signal intensity; y-axis: infected sample signal intensity. Red diagonal lines define areas of 2-fold differential signal intensities. Blue spots define unchanged expression; red dots: transcripts significantly upregulated and green dots: transcripts significantly down-regulated in the infected samples (p-value ≤0.01). The grey cross in the legend marks the number of significantly up- and downregulated genes. [file 1743-422X-10-61-S1.pdf]

**A**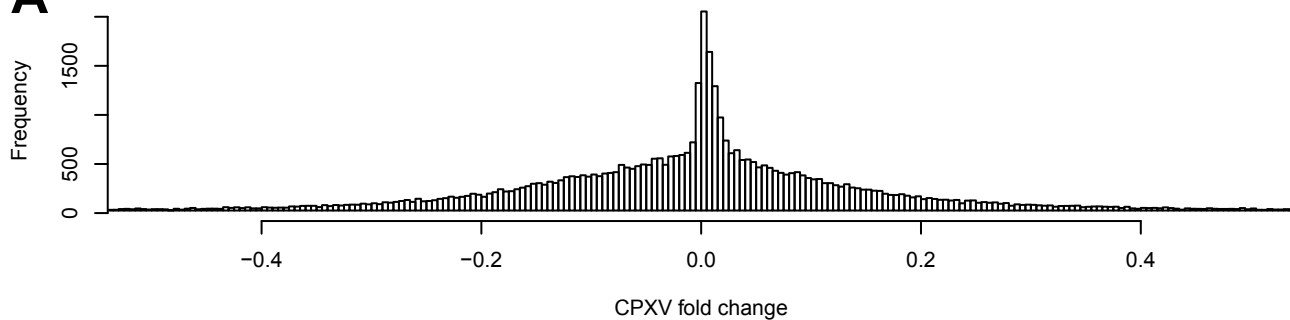**B**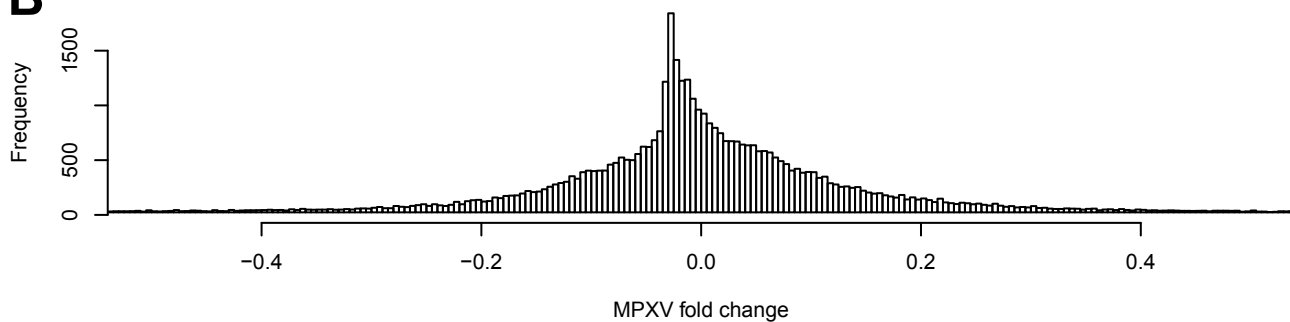**C**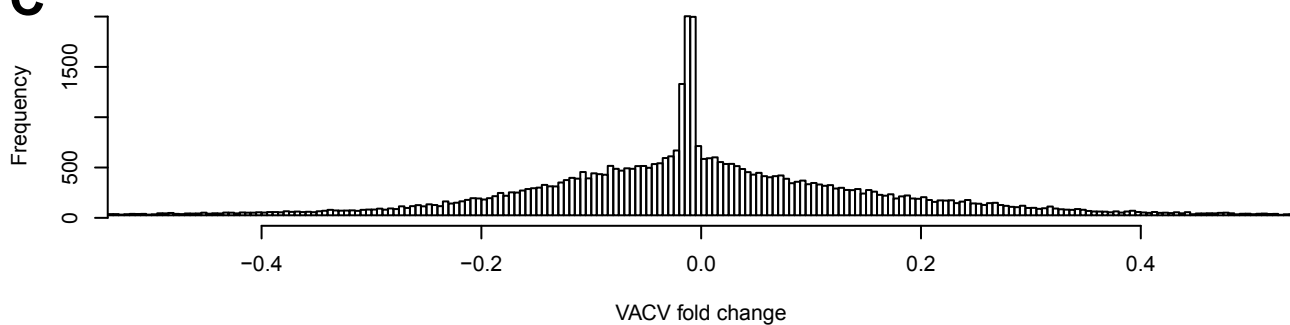

Supplement: Additional file 3 — Distribution of up- or downregulated genes. The figure shows the distribution of up- or downregulated transcripts following infection with CPXV (A), MPXV (B) or VACV (C) in comparison to non-infected cells. The degree of regulation compared to the non-infected control is displayed on the x-axis. A global minor downregulation below the set cut-off value of ≥2 fold change occurred following infection with MPXV and VACV. [file 1743-422X-10-61-S3.pdf]
