# Supplementary material for: Comparison of host cell gene expression in cowpox, monkeypox or vaccinia virus-infected cells reveals virus-specific regulation of immune response genes
Source: Virol J. 2013 Feb 20;10:61. doi: 10.1186/1743-422X-10-61 (PMC3599072; doi:10.1186/1743-422X-10-61)
Supplement: Additional file 2 — genes regulated by CPXV, MPXV and VACV. Shown is the relative expression of genes which were regulated by infection with all OPV tested to a similar extent. Included are genes that exhibited more than 2-fold up- or downregulation following infection. Red and green colour displays up- or downregulation in the infected cells compared to non-infected cells (shown are 2 arrays per virus). Numbers indicate the log10 of the change fold values. [file 1743-422X-10-61-S2.pdf]

| CPXV | | MPXV | | VACV | | Gene |
|---|---|---|---|---|---|---|
| 0.5 | 0.4 | 0.6 | 0.4 | 0.4 | 0.3 | EPHA2 |
| 0.5 | 0.5 | 0.6 | 0.3 | 0.4 | 0.3 | SPRED1 |
| 0.5 | 0.5 | 0.6 | 0.4 | 0.4 | 0.3 | ETV5 |
| 0.6 | 0.4 | 0.7 | 0.4 | 0.5 | 0.4 | RGS16 |
| 0.4 | 0.3 | 0.6 | 0.4 | 0.4 | 0.4 | PLK3 |
| 0.5 | 0.4 | 0.5 | 0.3 | 0.5 | 0.4 | U79293 |
| 0.6 | 0.6 | 0.6 | 0.5 | 0.3 | 0.3 | ZC3H12C |
| 0.6 | 0.4 | 0.6 | 0.5 | 0.4 | 0.3 | PHLDA1 |
| 0.7 | 0.6 | 0.4 | 0.3 | 0.4 | 0.4 | HBEGF |
| 0.8 | 0.6 | 0.7 | 0.5 | 0.8 | 0.5 | HS3ST1 |
| 0.7 | 0.5 | 0.7 | 0.3 | 0.7 | 0.6 | SPRED2 |
| 0.8 | 0.6 | 0.6 | 0.5 | 0.5 | 0.5 | ERRFI1 |
| 0.8 | 0.6 | 0.6 | 0.4 | 0.6 | 0.4 | DUOX1 |
| 0.5 | 0.4 | 0.6 | 0.8 | 0.5 | 0.4 | AF289567 |
| 0.4 | 0.5 | 0.6 | 0.6 | 0.4 | 0.5 | AU158345 |
| 0.5 | 0.4 | 0.7 | 0.8 | 0.6 | 0.7 | EGR2 |
| 0.5 | 0.8 | 0.4 | 0.7 | 0.5 | 0.8 | GFAP |
| 0.7 | 0.7 | 0.7 | 0.7 | 0.5 | 0.5 | THC2489251 |
| 0.7 | 0.7 | 0.7 | 0.7 | 0.5 | 0.5 | AW964144 |
| 0.8 | 0.7 | 0.8 | 0.7 | 0.4 | 0.4 | IL4R |
| 0.7 | 0.6 | 0.8 | 0.5 | 0.5 | 0.3 | TRIM15 |
| 0.6 | 0.6 | 0.7 | 0.7 | 0.4 | 0.4 | SMOX |
| 0.8 | 0.7 | 1 | 0.8 | 0.5 | 0.5 | LIF |
| 0.7 | 0.7 | 1 | 0.7 | 0.4 | 0.5 | THC2635964 |
| 0.8 | 0.7 | 0.9 | 1 | 0.5 | 0.5 | EREG |
| 0.6 | 0.6 | 0.7 | 1 | 0.3 | 0.3 | IER3 |
| 1 | 0.9 | 1.1 | 0.8 | 0.7 | 0.6 | AREG |
| 1.1 | 0.8 | 1.1 | 0.7 | 0.7 | 0.6 | MFSD2 |
| 0.9 | 0.9 | 1.2 | 0.7 | 0.6 | 0.7 | ARC |
| 1 | 0.7 | 1.3 | 0.7 | 0.9 | 0.6 | A_24_P698698 |
| 0.9 | 0.9 | 1 | 1 | 0.8 | 0.7 | DUSP6 |
| 0.9 | 0.9 | 1 | 0.8 | 0.8 | 0.8 | SPRY2 |
| 1.2 | 1 | 1 | 0.9 | 0.9 | 0.9 | MAFF |
| 0.9 | 0.7 | 1 | 0.5 | 0.8 | 0.7 | AB209345 |
| 0.8 | 0.5 | 0.9 | 0.3 | 0.9 | 0.7 | BC068044 |
| 0.6 | 0.6 | 1 | 0.5 | 0.8 | 0.8 | FOS |
| 0.7 | 0.6 | 0.8 | 0.3 | 0.9 | 1 | DNAJA4 |
| 0.9 | 0.8 | 0.8 | 0.6 | 0.6 | 0.5 | DUSP5 |
| 0.9 | 0.7 | 0.7 | 0.4 | 0.5 | 0.6 | EAF1 |
| 0.9 | 0.7 | 0.7 | 0.5 | 0.5 | 0.4 | THC2655811 |
| 1.1 | 1 | 0.8 | 0.4 | 0.4 | 0.5 | TBX2 |
| 1 | 0.9 | 1.1 | 0.4 | 0.5 | 0.4 | FUT1 |
| 1.2 | 1.2 | 0.6 | 0.6 | 0.7 | 0.7 | DNAJB3 |
| 1.4 | 1.4 | 1.2 | 1.8 | 0.5 | 0.5 | CXCL2 |
| 1.1 | 1.1 | 0.9 | 1.5 | 0.4 | 0.3 | PTGS2 |
| 1.7 | 1.1 | 2 | 1 | 1.4 | 0.8 | SPRY4 |
| 1.2 | 1.2 | 1.9 | 1.5 | 2 | 2.2 | EGR1 |
| 1.5 | 1.4 | 1.2 | 0.6 | 1.7 | 1.8 | AL833005 |
| 2.7 | 2.5 | 2.1 | 2.8 | 0.5 | 0.4 | CXCL1 |
| 0.3 | 0.3 | 0.3 | 0.3 | 0.2 | 0.2 | USP12 |
| 0.2 | 0.1 | 0.3 | 0.3 | 0 | 0 | HMGA2 |
| −0.1 | −0.1 | −0.1 | −0.2 | −0.1 | −0.1 | CSDC2 |
| −1 | −0.9 | −1.2 | −0.5 | −0.8 | −0.6 | BMF |
| −1.3 | −1.2 | −0.9 | −0.9 | −1.1 | −0.9 | AF086511 |
| −1.2 | −1 | −1.4 | −0.8 | −1.4 | −1.1 | EDN2 |
| −0.2 | −1.1 | −0.4 | −1.2 | −0.4 | −1.2 | ROS1 |
| −0.4 | −0.3 | −0.3 | −0.7 | −0.6 | −0.4 | THC2664573 |
| −0.3 | −0.6 | −0.4 | −0.5 | −0.4 | −0.5 | TRPM6 |
| −0.5 | −0.7 | −0.4 | −0.7 | −0.3 | −0.6 | ZBED2 |
| −0.2 | −0.1 | −0.4 | −0.3 | −0.5 | −0.4 | ADAMTS16 |
| −0.3 | −0.2 | −0.3 | −0.2 | −0.3 | −0.4 | DBP |
| −0.3 | −0.3 | −0.3 | −0.3 | −0.3 | −0.4 | AHNAK2 |
| −0.3 | −0.3 | −0.5 | −0.4 | −0.3 | −0.3 | CBLB |
| −0.4 | −0.4 | −0.3 | −0.3 | −0.3 | −0.3 | PRKCB |
| −0.5 | −0.4 | −0.4 | −0.4 | −0.2 | −0.3 | THC2656519 |
| −0.4 | −0.4 | −0.4 | −0.4 | −0.4 | −0.4 | VAV3 |
| −0.4 | −0.5 | −0.4 | −0.4 | −0.4 | −0.4 | LMO1 |
| −0.6 | −0.3 | −0.5 | −0.4 | −0.7 | −0.5 | KIAA1199 |
| −0.4 | −0.4 | −0.5 | −0.3 | −0.8 | −0.6 | CCDC85A |
| −0.6 | −0.3 | −0.5 | −0.4 | −0.5 | −0.6 | FLJ39609 |
| −0.6 | −0.5 | −0.6 | −0.3 | −0.6 | −0.4 | KCNQ1 |
| −0.5 | −0.5 | −0.5 | −0.3 | −0.5 | −0.6 | IFIT1 |
| −0.5 | −0.4 | −0.5 | −0.3 | −0.4 | −0.4 | KCNK3 |
| −0.6 | −0.5 | −0.5 | −0.4 | −0.5 | −0.4 | METTL7A |
| −0.8 | −0.9 | −0.9 | −0.8 | −0.5 | −0.5 | MARCH4 |
| −0.6 | −0.5 | −0.8 | −0.5 | −0.5 | −0.5 | PRR16 |
| −0.4 | −0.6 | −0.8 | −0.6 | −0.5 | −0.4 | DLC1 |
| −0.6 | −0.7 | −0.9 | −0.6 | −0.4 | −0.4 | AK024238 |
| −0.8 | −0.6 | −0.9 | −0.4 | −0.8 | −0.8 | IFIT2 |
| −0.7 | −0.8 | −0.6 | −0.5 | −0.7 | −0.7 | MAP1A |
| −0.8 | −0.9 | −0.7 | −0.4 | −0.6 | −0.6 | A_24_P290087 |
| −0.8 | −0.5 | −0.6 | −0.6 | −0.5 | −0.7 | BC037328 |
| −0.6 | −0.6 | −0.6 | −0.7 | −0.6 | −0.6 | HECW2 |
| −0.6 | −0.6 | −0.6 | −0.5 | −0.5 | −0.5 | FOXO4 |
| −0.6 | −0.6 | −0.6 | −0.5 | −0.6 | −0.5 | PROC |
| −0.7 | −0.7 | −0.6 | −0.5 | −0.5 | −0.5 | CPA4 |
| −0.7 | −0.7 | −0.6 | −0.5 | −0.5 | −0.5 | PLCXD3 |
